# Efficacy and Safety of Thalidomide for Chemotherapy-induced Nausea and Vomiting

**DOI:** 10.7150/jca.45678

**Published:** 2020-05-18

**Authors:** Nan Wang, Peng Xu, Yu Liu, Peng Zhao, Jian Ruan, Yi Zheng, Junpei Jin, Shuqian Wang, Jia Yao, Dong Xiang, Dai Zhang, Na Li, Huafeng Kang, Zhijun Dai

**Affiliations:** 1 Department of Breast Surgery, The First Affiliated Hospital, College of Medicine, Zhejiang University, Hangzhou, China.; 2 Department of Oncology, The Second Affiliated Hospital of Xi'an Jiaotong University, Xi'an, China.; 3 Department of Medical Oncology, The First Affiliated Hospital, College of Medicine, Zhejiang University, Hangzhou, China.; 4 Celilo Cancer Center, Oregon Health Science Center affiliated Mid-Columbia medical center, The Dalles, OR, USA.

**Keywords:** Thalidomide, Chemotherapy-induced nausea and vomiting, Emesis

## Abstract

**Purpose**: A substantial number of cancer patients discontinue chemotherapy due to severe chemotherapy-induced nausea and vomiting (CINV). This study aimed to evaluate the efficacy and safety of thalidomide (THD) in CINV.

**Methods**: We searched different databases to identify related studies that investigated the efficacy and safety of THD in CINV. The primary outcomes were CINV in the acute (0-24 h), delayed (24-120 h), and overall (0-120 h) phases, respectively. The secondary outcomes were the safety of THD and the patients' quality of life (QOL).

**Results**: Fourteen randomized control trials (RCTs) including 1744 patients (42% male) reported the risk ratio (RR) and 95%CI of the THD group versus control group in reducing nausea and vomiting. Meta-analysis showed that THD statistically enhanced the complete response rate of nausea and vomiting in the delayed (nausea: RR = 1.69, 95%CI: 1.47-1.94; vomiting: RR = 1.38, 95%CI: 1.26-1.51) and overall phases (nausea: RR = 1.54, 95%CI: 1.31-1.81; vomiting: RR = 1.31, 95%CI: 1.18-1.46). Furthermore, subgroup analysis based on THD dosage (100 *vs* 200 mg/day) demonstrated no statistical significance with respect to overlapping 95%CI. Thirty studies monitored the adverse events (AEs) of THD, all under grade 3 based on the CTCAE criteria. We compared the eight most common AEs; sedation, constipation, and drowsiness/dizziness were slightly frequent compared with controls.

**Conclusion**: THD is an effective adjuvant and a potential alternative in reducing delayed and overall CINV. Other regimens might be added for CINV during the acute phase.

## Introduction

Chemotherapy-induced nausea and vomiting (CINV) is caused by neurotransmitters and chemical substances stimulating the receptors in either the vomiting center or the chemoreceptor trigger zone. These substances include dopamine, serotonin, histamine, acetylcholine, and substance P (NK1) 1-4. CINV needs to be well controlled because it often poses difficulties in chemotherapy, making it hard to maintain dose intensity and consequently reducing the patients' quality of life (QOL) 5. The occurrence of CINV has been evaluated to be as high as 70-80% without proper antiemetic regimens 6. Therapy has evolved considerably over the past four decades; the most recognizable and followed guidelines now recommend a four-drug combination including NK1 receptor antagonist, 5-HT3 receptor antagonist, dexamethasone, and olanzapine to prevent CINV in high emetic chemotherapy (HEC)^7^, ^8^. However, 60-80% of patients still experience CINV alongside chemotherapy 9, and the high cost of the present treatments for CINV also limit their clinical practice and promotion to an extent.

Thalidomide (THD) is a derivative of glutarnic acid, which was initially used as a sedative to treat emesis in pregnancy but was withdrawn from the market as it caused a serious adverse reaction to the fetal seal. THD could attenuate cisplatin-induced delayed emesis and decrease the levels of NK1 in the medulla and gastric tissues in a rat model 10. In 2009, Liu et al. initially reported its significant effects in preventing chemotherapy-induced gastrointestinal side effects in the delayed phase following a modified FOLFOX7 regimen 11. THD is also a powerful immunomodulatory and antiangiogenic drug that can inhibit the expression of vascular endothelial growth factor proteins and induce cell apoptosis 12. The US food and drug Administration approved it as a treatment for multiple myeloma. Current studies have shown that chemotherapy combined with THD can be applied to treat solid carcinomas including lung cancer, breast cancer, gastric cancer, rectal cancer, and pancreatic cancer with a prominent curative effect 13-17. A report also showed that THD could alleviate the symptoms accompanying malignant tumors, including cachexia, chronic nausea, insomnia, cancer pain, and dysesthesia 18.

Studies in succession have reported the notable effects of THD on CINV prevention during chemotherapy in patients with solid tumors in recent years. However, neither a systematic review nor a meta-analysis has been conducted based on the current progress. Therefore, we collected the studies related to THD in reducing nausea and emesis in chemotherapy patients and conducted an integrated analysis based on the currently available studies to see if it is an adjuvant for the currently recommended anti-CINV drugs or a potential alternative for antipsychotic or hormonal drugs for patients who cannot tolerate them.

## Methods

### Search strategy

A comprehensive literature search of all publication years up to Nov.30th 2019 was performed in PubMed, Embase, Cochrane, Web of science, CNKI, and Wanfang. The website of clinicaltrials.gov was searched for unpublished studies. Keywords related to intervention (“thalidomide” OR “sedoval” OR “thalomid” OR “N-phthaloylglutamic acid”) were combined with keywords related to therapy (“chemotherapy*” NOT "radiotherapy*”) and terms related to CINV (“chemotherapy-induced nausea and vomiting” OR “CINV” OR “nausea” OR “vomit” OR “emesis” OR “gastrointestinal side effect” OR “gastrointestinal dysfunction”). Furthermore, the reference lists of all searched studies were also taken into consideration.

### Study selection and criteria

We evaluated the study eligibility with the PICO approach (population, intervention, comparison, and outcome). Only eligible randomized controlled trials (RCTs) contributed to the primary outcome assessment. Studies ineligible for the primary outcome but specifying the safety and QOL outcomes were included in analyzing the secondary outcomes.

Population: Patients received chemotherapy (e.g. cisplatin, oxaliplatin, nedaplatin). Any form of radiotherapy-involved treatment (e.g. Concurrent chemo-radiotherapy) was excluded.

Intervention: THD was used as an add-on treatment based on some regular anti-CINV regimen: 5-HT3 RA with or without dexamethasone/methylprednisolone/metoclopramide.

Comparison: Eligible studies were required to apply the same regimen except for THD as the control group. Case studies, studies including two anti-CINV groups and no control group, and open trials without controlled pre-post designs were eliminated from the meta-analysis.

Outcome: Ranked data of nausea or vomiting degrees, or incidence rate of nausea or vomiting in the acute, delayed, or overall phase, which could be converted to the complete response rate were included. Studies providing effective response only, which equals the complete plus partial response, combining nausea and vomiting as a single outcome, were excluded.

### Quality assessment and data extraction

Two investigators (N W and P X) independently reviewed the included studies and extracted relevant data with a prespecified table (Table 1). Extracted information included year of publication, first author, sample size, and subject characteristics (such as mean ages, cancer types, treatment, and comparability of QOL). The overall quality of included studies analyzing the primary outcome was assessed according to the criteria for bias risk assessment in the Cochrane collaboration handbook 5.1.4^19^. All eligible trials reported the application of randomization. Among these, two studies mentioned the method of randomization 20, 21: one used a computer-generated sequence 20, another used a random number table 21; the remaining studies did not report any details of randomization. None of the studies reported whether the treatment allocation was concealed except for one that used identical capsules 20. Five studies 22-26 used a double-blinding method and one 20 used a triple-blinding method in the experimental process. Studies presenting the data with endpoints of subjects and baseline characteristics were regarded as reporting complete data. We further differentiated “other sources of bias” with three subdomains: “enrolment” (e.g. ratio of total attended to planned participants), adherence (e.g. ratio of planned therapy cycles successfully finished with the planned dosage in cycles attended), and loss to follow-up (e.g. uneven dropouts between the intervention and control groups). The detailed information about quality evaluation in each study is presented in Figure 2.

### Outcomes of interest

The primary endpoint was the rate of complete response (CR) for nausea and vomiting in the acute, delayed, and overall phases. The secondary endpoint was the safety of THD, which was assessed based on the common terminology criteria for adverse events (CTCAE) and patients' QOL changes, assessed by Karnofsky Performance Status (KPS) scores.

### Statistical analysis

For the primary endpoint, studies were stratified by the reaction phases (acute, delayed, overall) of prognosis (nausea and emesis). Pooled risk ratios (RRs) with 95%CI weighted by the Mantel-Haenszel method in the fixed model were used to calculate the difference of CR between the THD and control groups. The difference was tested with α = 0.01. Subgroup analysis was performed based on the THD dosage (100 *vs* 200 mg/day) to investigate different therapeutic effects. The patients' QOL was estimated by improved rates of KPS scores, and the safety of THD was calculated by the pooled odds ratio (OR) in the fixed model.

Heterogeneity was investigated by I² and Q statistics 27. A more liberal *P* value of ≤ 0.10 was referred to signify heterogeneity, considering the generally low statistical power of heterogeneity tests 28. The I² statistic is an estimate of variance in a pooled effect size, which is explained by heterogeneity in the study samples and is unaffected by the study quantities (K) 29. Values of 0%, 25%, 50%, and 75% were determined to indicate no, low, moderate, and high heterogeneity, respectively.

Negative and positive findings are partially published to some extent, and publication bias is a widespread problem when reviewing the available references 30. We assessed publication bias using funnel plots and Egger's test 31-33. If the results suggested possible publication bias, adjusted ESs were computed with the Duval and Tweedie trim-and-fill method 34. We calculated a failsafe number in case of statistically significant results 33, 35. This failsafe number is the number of unpublished studies without findings, which would reduce the results to statistical non-significance (*P* > 0.05). We also tested the robustness of the results by comparing the suggested criterion (5K + 10) to the failsafe number 35.

## Results

### Characteristics of included studies

**Figure 1:**
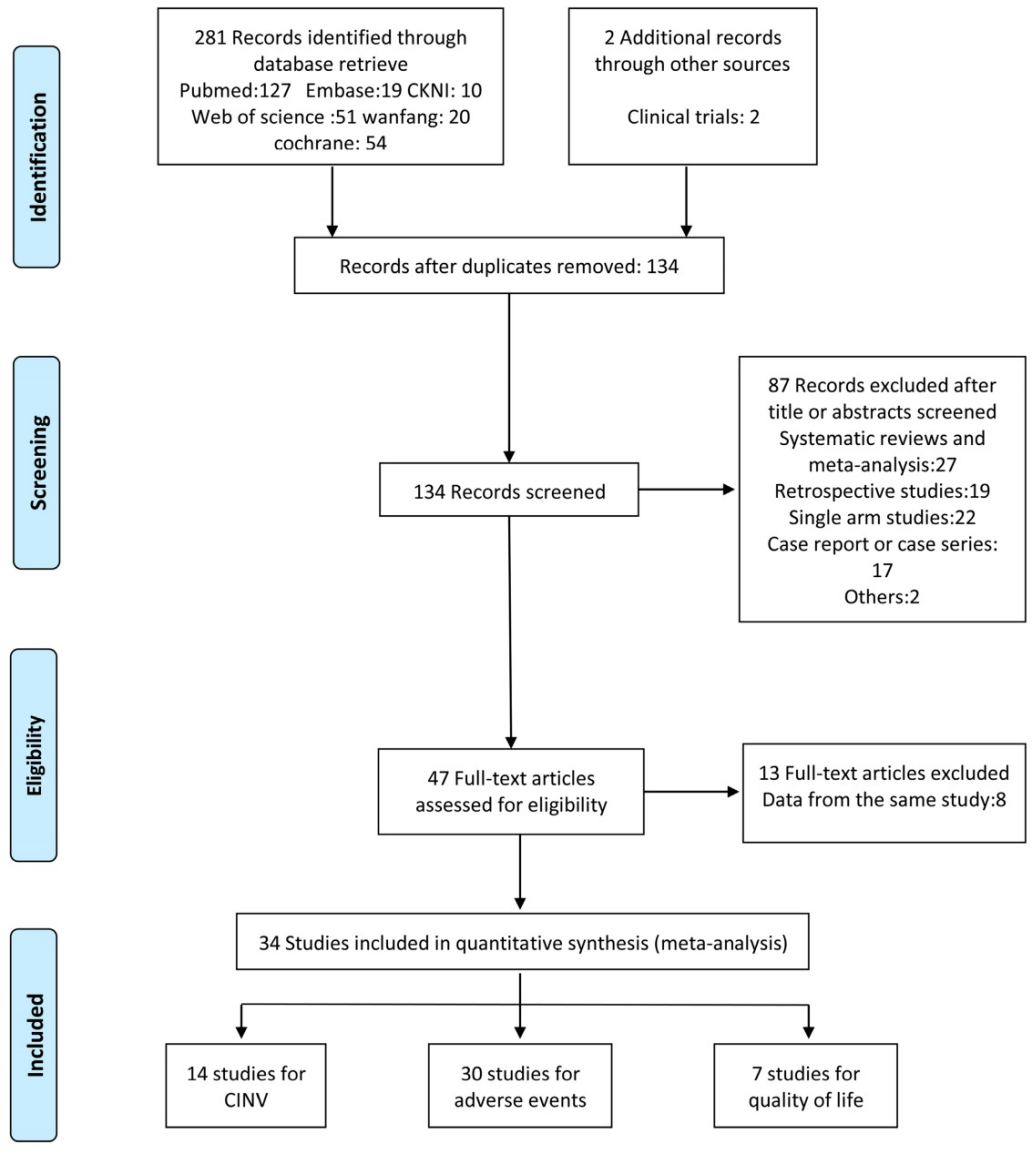
Study selection flowchart.

**Figure 2:**
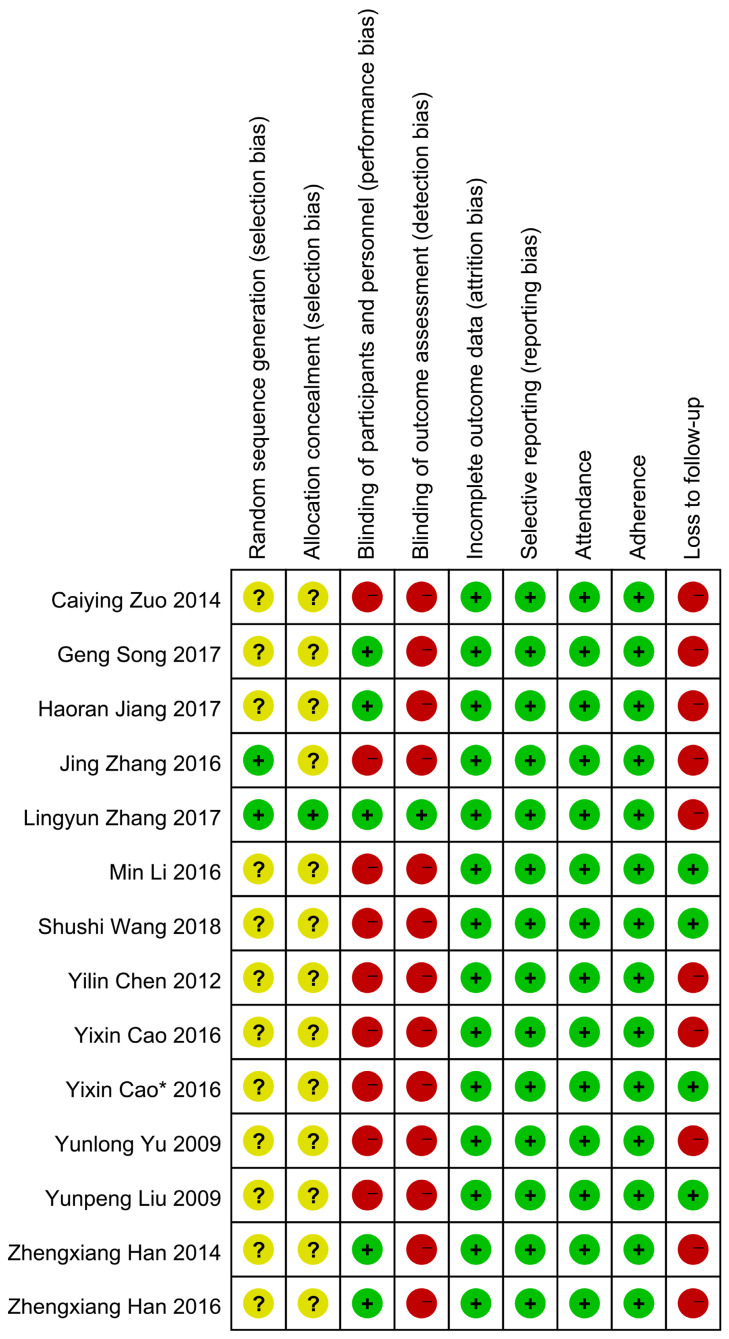
Risk of bias. Notes: Green cycle with plus sign indicates a low risk of bias; Yellow cycle with question mark indicates an unclear risk of bias; Red cycle with minus sign indicates a high risk of bias.

In total, 283 studies were identified by screening the six databases mentioned above. We excluded 149 duplicates and reviewed the abstracts of the remaining 134 studies based on our inclusion and exclusion criteria. 47 studies met the eligibility criteria and were inspected further. We found that 13 of them were from duplicate population. Another 20 studies were combined in the secondary outcome analysis. Among these, 16 studies were excluded from the primary outcome analysis as they combined nausea and emesis as a single outcome or offered indefinite data (e.g. unable to extract complete response measurement). 4 studies were eliminated for comparison with positive control (e.g. dexamethasone/metoclopramide). Finally, 14 RCTs were subjected to our first endpoint meta-analysis. The flowchart of the study selection is shown in Figure 1.

The characteristics of the included studies are detailed in Table 1. The number of patients in the 14 RCTs varied from 52 to 638. Overall, 877 (50%, mean standard deviation age: 55 ± 5.5 years) and 867 (50%, mean standard deviation age: 56 ± 4.4 years) patients allocated to THD and control groups, respectively, were evaluated. The CR data of nausea and vomiting in the three phases were extracted from two studies 20, 22, with one presenting the original data on five separate days and over the entire study duration 22. We selected the smallest records from days 2-5 of the two groups as the relative conservative indicator for the patient number that achieved CR in the delayed phase. One study offered the rates of CR for nausea in the three phases 23. The CR data for nausea and vomiting in the acute and delayed phases were extracted from four studies 21, 36-38 with one giving the original data on five separate days 38. Smallest records from days 2-5 of the two groups were extracted respectively to indicate the patient number that achieved CR in the delayed phase. One study reported the rates of CR for nausea and vomiting in the acute phase 11. Three studies reported the rates of CR of nausea and vomiting in the overall phase 26, 39, 40. Two studies reported the rate of CR for vomiting in the overall phase 24, 41, and one in the delayed phase 25. Four of the included studies 24-26, 40 were from two same authors but were four independent trials with different patient characteristics, anti-CINV regimens, and cared outcome phases. Most studies used platinum drug-based combined chemotherapy, such as cisplatin, oxaliplatin, and nedaplatin of which cisplatin was most used.

### Primary endpoint (no nausea and no vomiting)

**Figure 3:**
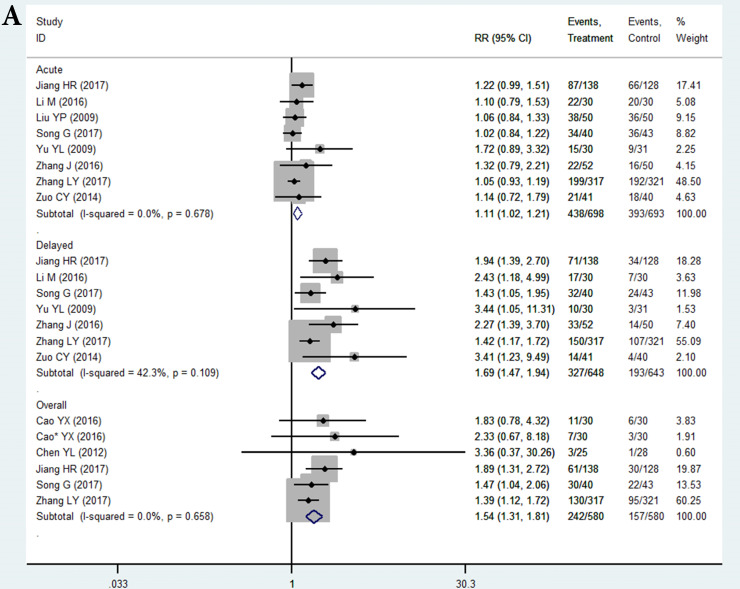

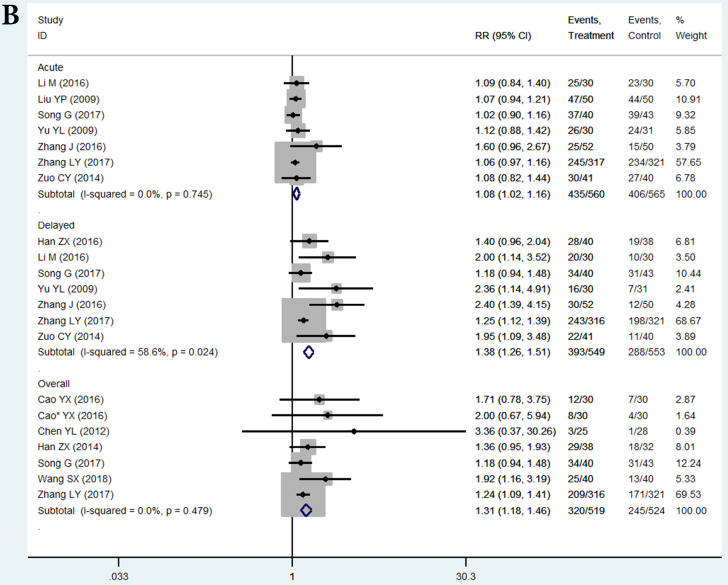
A. Forest plot of effect sizes for effects of thalidomide on anti-chemotherapy induced nausea in the three phases (acute, delayed and overall). B. Forest plot of effect sizes for effects of thalidomide on anti-chemotherapy induced vomiting in the three phases (acute, delayed and overall).

In the selected 14 RCTs, the CR for nausea in the THD group did not significantly differ from that in the control during the acute phase (RR = 1.11, 95%CI: 1.02-1.21, *P* > 0.01; Figure 3A). The positive result of RR with the lower limit of 95%CI extremely close to 1 may be attributed to sampling error, since none of the studies in the acute phase showed a positive result. However, patients in the THD group showed a statistically better CR during both the delayed (RR = 1.69, 95%CI: 1.47-1.94, *P* < 0.01 Figure 3A) and overall phase (RR = 1.54, 95%CI: 1.31-1.81, *P* < 0.01 Figure 3A) compared to the control group.

The CR for chemotherapy-induced vomiting in the THD group did not significantly differ from that in the control during the acute phase (RR = 1.08 95%CI: 1.02-1.16, P>0.01 Figure 3B). We did not impart much clinical meaning to the pooled RR as well as the result of acute nausea. However, patients in the THD group had a statistically positive CR during both the delayed (RR = 1.38, 95%CI: 1.26-1.51, *P* < 0.01 Figure 3B) and overall phase (RR = 1.31, 95%CI 1.18-1.46, *P* < 0.01, Figure 3B) compared with the control.

**Figure 4:**
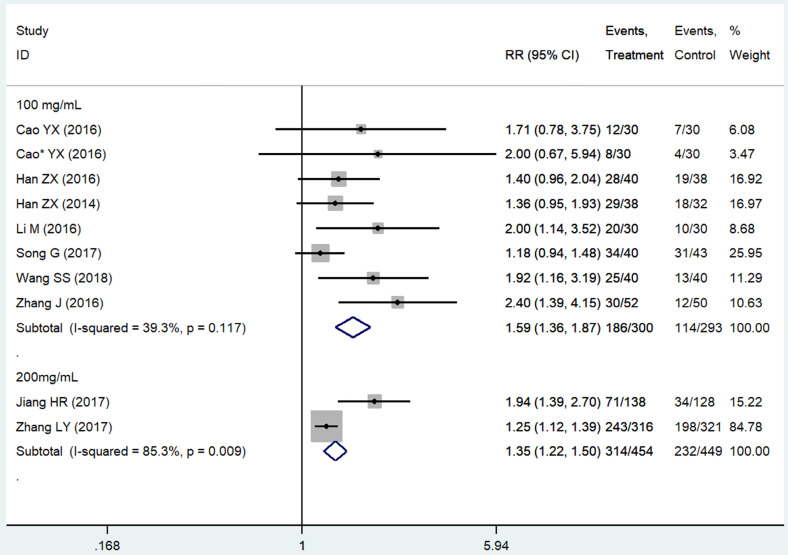
Forest plot of effect sizes for effects of subgrouped thalidomide dosages (100 vs 200 mg/d) on CINV in the delayed phase.

Subgroup analysis based on the THD dosage was conducted. We extracted all data on delayed vomiting (for its rather high heterogeneity), overall vomiting data (studies 24, 26, 40, 41 only provided the overall vomiting data were used as an indicator of the delayed phase ones), and one case of delayed nausea (vomiting was not an outcome in that study 23). Outcomes from acute phase were not considered in view of the limited effect, which might compromise the statistical power. Emetic chemotherapies of the 10 studies were all cisplatin-contained regimens, which was HEC. In those studies, THD was used in the range of 50-200 mg/day, and 100 and 200 mg/day were mostly used. The 95%CI of pooled RR was compared between the effect of 100 mg/day (RR = 1.59, 95%CI 1.36-1.87, *P* < 0.01; Figure 4) and 200 mg/day (RR = 1.35, 95%CI 1.22-1.50, *P* < 0.01; Figure 4) of THD. The overlapping of 95%CI meant no statistical difference.

### Publication bias

**Figure 5:**
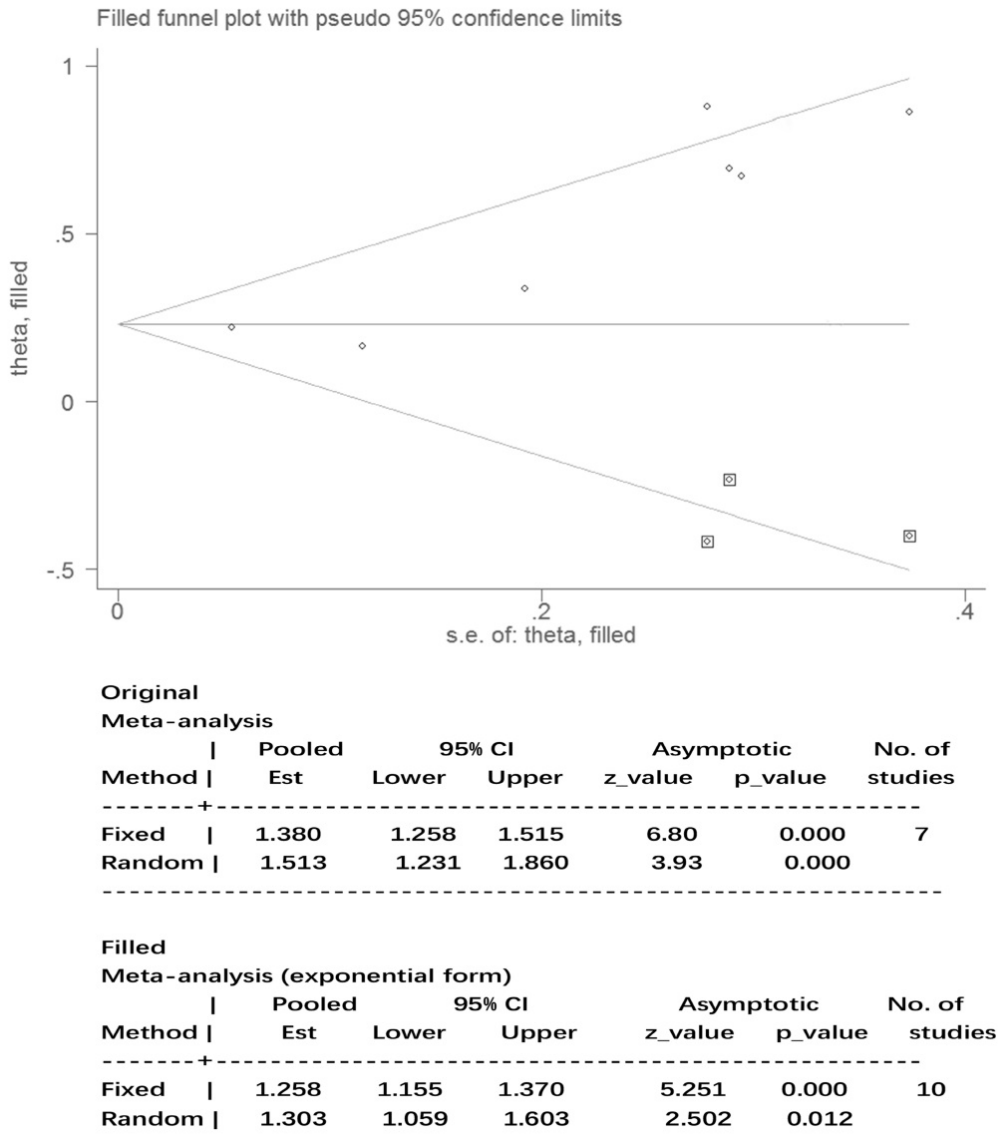
Publication bias after Duval and Tweedie trim-and-fill method. Abbreviations: Est: estimates

The results for nausea in the three phases and vomiting in the acute and overall phase were regarded as statistically homogeneous (I² < 50% *P* > 0.1). Statistical heterogeneity (I² = 58.6% *P* = 0.02) within the delayed vomiting group was shown by the Egger's test, which was used to evaluate the publication bias (*P* = 0.01, 95%CI: 1.05-5.23). The adjusted summary estimate was calculated using the Duval and Tweedie trim-and-fill method, the SE hardly changed after three supplementary studies as shown in Figure 5. Therefore, publication bias did not affect the stability of the outcome. The Rosenthal failsafe number 104 was also calculated and outdistanced the suggested criterion: 45 (5K + 10), which testified a robust result.

### Safety of thalidomide

No grade 3 or 4 side effects were reported in the THD group in the 39 studies according to the CTCAE criteria. Sedation was the most common adverse event reported followed by constipation and drowsiness/dizziness. It seemed that THD was relatively safe except that the rate of peripheral neuropathy (*P* = 0.06) differed significantly between the THD and control groups as shown in Table 2.

### Quality of life (QOL)

**Figure 6:**
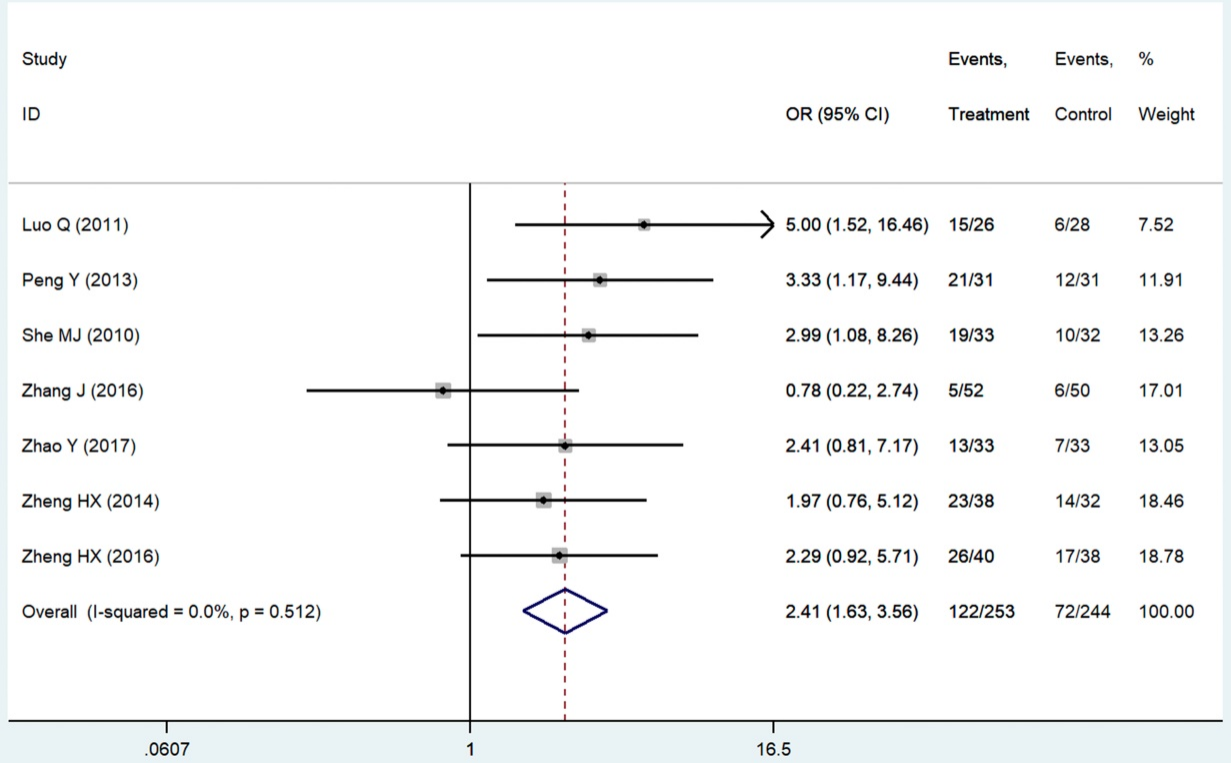
Forest plot of effect sizes for effects of thalidomide on patients' quality of life.

The evaluation indexes of patients' QOL were insufficient and non-standard. We pooled six improved rates of KPS scores 14, 21, 24, 25, 42, 43, which were the mostly used standards in our related studies and one ECOG score 44 converted to that. The result was significant (RR = 2.41 95%CI: 1.63-3.56; *P* < 0.01, Figure 6). Criteria like SAS & SDS 26, QOL questionnaire C30 by European Organization for Research and Treatment of Cancer 20 and CAT 18 also showed statistically better results of the patients' appetite, sleep quality and emotion in the THD group as well as patients' sensation of wellbeing 45.

## Discussion

This meta-analysis investigated the add-on prophylactic treatment potential of THD for CINV. Complete response rates of nausea and vomiting were significantly higher in the THD-treated group in the delayed and overall phases than the acute phase. As for studies were not included in our analysis for comparison with non-blank group (e.g. metoclopramide/dexamethasone) 44, 46-48, the THD group also showed a statistically better control of nausea and emesis in the delayed phase. In most of our included studies, THD was administered at 50-200 mg/day, with 100 mg/day being the most common. Subgroup analysis based on the dosage suggested no statistical significance between the 200 mg/day dose compared with the 100 mg/day dose. We thus suggest the dose of 100 mg/day of THD for prophylaxis of CINV in consideration of adverse events (AEs). Nevertheless, this recommendation is speculative, given an incomprehensive dose gradient and the small number of pooled trials.

Essentially, risks related to THD today remain the same as those when it was originally produced and marketed in more than 45 countries, nearly 70 years ago. The overall side effects of THD are well understood as different patient populations have been exposed for decades of clinical application. However, chemotherapy patients do not bear the risk of birth defects. Other common AEs of THD were estimated by pooled OR in a fixed-effect model. Sedation, constipation, and dizziness/drowsiness were the most common side effects (Table 2). Nevertheless, AEs did not bring about level 3 or 4 toxicity and could be tolerated by patients. No other THD-related AEs were suggested in our analysis. However, the* P* value (0.06) of peripheral neuropathy should be noted. This complication may be associated with cumulative dosage, emphasizing the requirement of defined recommendations for monitoring, tapering, and discontinuation. Though 18 studies we totally collected specifically targeted on the investigation of THD on CINV, there were no high quality RCTs further comparing THD and some regular drugs such as: NK1-RA, Olanzapine or Dexamethasone recommended in the Guidelines of American Society of Clinical Oncology (ASCO) and National Comprehensive Cancer Network (NCCN)^9^. Well-designed clinical trials between THD and these anti-CINV drugs are required to line THD up to a waitlist for the antiemetic treatment scenario.

THD, as an antiangiogenic agent, has produced substantial clinical benefits in the treatment of multiple myeloma, and its effects on a host of solid tumors have been quite variable. There seems to be a reasonable consensus that THD has demonstrable tumoristatic effects in renal cell carcinoma 49, hepatocellular carcinoma 50, 51, prostate carcinoma 52, Kaposi's sarcoma 53, melanoma 54, glioma 55, and glioblastoma multiforme 56. The main pathophysiological features of advanced cancer are insomnia, chronic emesis, nausea, cachexia, metabolic disorders, and tumor-associated pain as well as decreased sensation of wellbeing. Perhaps the most intriguing quality of THD is its underlying value in many of these syndromes, as demonstrated in our analysis, and the fact that it is well tolerated in this very ill patient population in general. Emotional disturbance is quite usual after cancer diagnosis, and it could adversely affect treatment, sleep, and appetite. We observed that THD enhanced the CR of CINV as well as improved the diet and sleep of patients compared to those on a non-THD regimen 20. Bruera et al. 45 reported a significant increase in caloric intake for 27 patients who were able to complete their food intake, from 1320 calories on day 0 to 1531 calories on day 10 (*P* = 0.047). In the THD group, 93% of patients hoped to take THD again in the next cycle of chemotherapy. Better chemotherapy compliance was noted in the THD group. The latent role of THD within the field of palliative treatment thus requires further investigation.

Elucidation of the heterogeneity of the effect in the delayed vomiting phase is critical to inform an individualized therapy and precision medicine approach, one that encompasses chemotherapy and patients' characteristics to guide targeted THD prescriptions. For example, when CINV patients also develop insomnia, diarrhea, irritability, or hyperactivity, a corresponding dosage of THD may be a more appropriate choice. THD has synergistic and additive antiemetic, anti-asthenia, and analgesic effects of corticosteroids 57, 58; it inhibits the expression of cytokines without affecting levels of IL-2, IL-4, and IL-10 59, 60, thus offering the possibility of steroid-sparing or steroid-replacement therapy. Rigorous examination and enforcement of such an approach pose significant challenges to the field but could hold great promise to improve the safety and efficacy of anti-CINV therapy in the clinical environment.

The strengths of our analysis are as follows. First, the pooled studies were all RCTs that enhanced the evidence grade. Second, the control group were all uniformed to blank controls, which eliminated some confounding factors to an extent. Third, we investigated the safety and QOL outcome as we explored the efficacy of THD. However, objective limitations still exist. Firstly, an insufficient number of studies used moderately emetogenic chemotherapy which limited our further investigation of specifying THD's role in moderate and high categories in anti-CINV. Secondly, one study with 638 patients outnumbered other studies in population may cause some inevitable bias. Lastly, all the included patients were Chinese; thus, generalizability of the recommended dosage to other ethnic groups requires caution.

In conclusion, THD is an effective adjunctive treatment to improve CINV in patients receiving emetic chemotherapy according to our results. Well-designed clinical trials are required to compare the efficacy and safety between THD and NK1-RA or olanzapine to define the place of THD in therapy for the prophylaxis of CINV, to consider the combination of these medicines as the optimum choice, and to assess the AEs systematically. THD is indeed worth considering for prophylactic antiemetic treatment.

## Figures and Tables

**Table 1:** Baseline characteristics of 34 studies for meta-analysis

First author	Study design	Number			Sexes			Age		Cancer Types	Therapeutic Regimen	Antiemetic Regimen	QOL before treatment	Dosage (mg/d)	Outcomes
		T+C	C	Male		Female		T+C	C						
Cui Y 2011	RCS	26	26		0	56		NR		B	AC-T	Tro+THD VS Tro+DEX	ECOG PS 0-2	25X2	AEs
Chen YL 2012	RCT	25	28		31	22		58.1	57.9	L+C+B	Cisplatin-based	Tro+THD VS Tro	KPS≥70	50x3	Anti-nausea and vomiting(O)
Cao YX 2016	RCT	30	30		31	29		45.98	50.53	L+C+B	Cisplatin-based	PAL+THD VS PAL	ECOG PS 0-2	100x1	Anti-nausea and vomiting(O)
Cao YX* 2016	RCT	30	30		35	25		68.25	64.6	L+G+C	NR	PAL+THD VS PAL	NR	100x1	Anti-nausea and vomiting(O)
Cheng QL 2018	RCS	45	45		0	90		54.91	55.09	C*	Cisplatin-contained	PAL+THD VS PAL	NR	50x1	AEs
Feng G 2015	RCT	35	35		25	20		58	57	L+E+O	NDP-contained	Aza+THD VS Aza	KPS 95 96	200x1	AEs
Gu AQ 2009	RCS	33	33		20	21		56	54	NSCLC	NP	THD VS placebo	ECOG PS 0-2	200x1	AEs
He QS 2008	RCT	21	20		20	21		56	54	NSCLC	NP	THD VS placebo	ECOG PS 0-2	200	AEs
Han ZX 2014	RCT	38	32		40	30		50		L+G+O	Cisplatin-contained	Tro+THD VS Tro	KPS>60	100-200	Anti-vomiting(O);QOL
Han ZX 2016	RCT	40	38		45	33		50.4	50.2	L+G+O	Cisplatin-contained	Aza+TDH VS Aza	KPS≥60	100-200	Anti-vomiting(D);AEs;QOL
Jiang WM 2010	CCS	31	30		40	21		56	57	NSCLC	GP	THD VS placebo	ECOG PS 0-2	200x1	AEs
Jiang HR 2017	RCT	138	128		94	172		59.4	59.5	L+B	CE or cisplatin-contained	PAL+DEX+THD VS PAL+DEX	ECOG PS 0-2	100X2	Anti-nausea(A;D;O);AEs
Luo Q 2011	RCS	26	28		42	12		60	59	NSCLC	GP	THD VS placebo	KPS≥70	100-200	QOL
Liu YP 2009	RCT	26	26		35	17		55.5	54	G+C+O*	mFOLFOX7	RAM+DEX+THD VS RAM+DEX	ECOG PS 0-2	150X2	Anti-nausea and vomiting(A); AEs
Li M 2016	RCT	30	30		35	25		56.8	57.7	L	Cisplatin-contained	OND+DEX+THD VS OND+DEX	ECOG PS 0-2	100x1	Anti-nausea and vomiting(A;D);AEs
Peng Y 2014	RCT	31	31		38	24		68.5	69.7	NSCLC	TP	5-HT3 RA+THD VS 5-HT3RA	KPS≥60	100-200	AEs; QOL
Qv H 2018	CCS	47	47		0	94		46.81	47.52	O	TC	Tro+THD VS Tro	KPS≥70	50x1	AEs
Shen ZL 2009	CCS	26	10		22	14		46.8	45.2	NSCLC	NP	THD VS placebo	ECOG PS 0-2	100-400	AEs
Song XQ 2010	RCS	35	31		52	14		56	55	NSCLC	Cisplatin-based	THD VS placebo	ECOG PS 0-2	300x1	AEs
She MJ 2010	RCT	33	32		44	21		NR		E	FP	OND+Met+THD VS OND+Met	KPS≥70	100-200	AEs; QOL
Sun YL 2010	CCS	36	21		31	26		54	52	NSCLC	NP	THD VS placebo	ECOG PS 0-2	200x1	AEs
Song XG 2010	RCS	35	31		52	14		56	55	NSCLC	Cisplatin-based	THD VS placebo	ECOG PS 0-2	300x1	AEs
Sun XQ 2011	CCS	30	30		36	24		57.5		NSCLC	DP	THD VS placebo	ECOG PS 0-2	300x1	AEs
Song G 2017	RCT	40	43		57	26		57	54	G+L+E	FP or EP	OND+MET+DEX+THD VS OND+MET+DEX	KPS 70-100	100x1	Anti-nausea and vomiting(A;D;O);AEs
Wang SS 2018	RCT	40	40		55	25		52.21	51.47	L	Cisplatin-contained	PAL+DEX+THD VS PAL+DEX	ECOG PS 0-1	100x1	Anti-vomiting(O)
Xu SN 2010	CCS	30	30		32	28		55		E	PTX+NDP	THD VS Placebo	ECOG PS 0-1	100-300	AEs
Yu YL 2009	RCT	30	31		29	32		58	62	NSCLC	GP	Ram+Met+THD VS Ram+Met	ECOG PS 1 1	50x2	Anti-nausea and vomiting(A;D)
Zhang GJ 2008	RCT	30	30		39	21		57		NSCLC	DP	THD VS placebo	ECOG PS 0-2	300	AEs
Zhu ZT 2010	RCS	40 40			50	30		48		NSCLC	GP	RAM+DEX+THD VS RAM+DEX+Met	NR	50x2	AEs
Zuo CY 2014	RCT	41	40		0	82		55	57	MBC	GP	Tro+THD VS Tro	KPS 60-90	25x2	Anti-nausea and vomiting(A;D);AEs
Zhang J 2016	RCT	52	50		58	44		NR		SCLC	EP or IP	PAL+MP+THD VS Tro+MP	KPS≥70	100x1	Anti-nausea and vomiting(A;D);AEs;QOL
Zhao W 2016	RCS	39	39		42	36		57.2		NR	Cisplatin-contained	Tro+DEX+THD VS Tro+DEX	NR	25x2	AEs
Zhao Y 2017	RCS	33	33		36	30		57.5		L	GP	THD VS Tro	ECOG PS 0-2	100x1	QOL
Zhang LY 2017	RCT	317	321		195	443		53	54	L+B+O*	CE or cisplatin-contained	PAL+DEX+THD VS PAL+DEX	ECOG PS 0-2	100x2	Anti-nausea and vomiting(A;D;O);AEs

Abbreviations: RCT: Randomized Clinical Trials; RCS: Retrospective Cohort Studies; CCS: Case Control Studies; T+C: Thalidomide group; C: Control group; L: Lung cancer; B: Breast cancer; G: Gastric cancer; C: Colorectal cancer; C*: Cervical cancer; O: Ovarian cancer; O*: Others; E: Esophageal cancer; NSCLC: Non-small cell lung cancer; SCLC: Small cell lung cancer; MBC: metastatic breast cancer; CE: Carboplatin+VP16; mFOLFOX7: Oxaliplatin+ Calcium Folinatc+ Fluorouracil; EP: VP16+ Cisplatin; GP: Gemcitabine+ Cisplatin; IP: Irinotecan+ Cisplatin; TC: Paclitaxel+ Carboplatin; AC-T: Anthracycline+ Cytotoxic agent-Taxol drugs; PTX: Paclitaxel; NDP: Nedaplatin; TP: Paclitaxel+ Cisplatin; FP: Fluorouracil+ Cisplatin; DP: Docetaxel+ Cisplatin; NP: Vinorelbine+ Cisplatin; Tro: Tropisetron; Aza: Azasetron; RAM: Ramosetron; DEX: Dexamethasone; PAL: Palonosetron; Met: Metoclopramide; MP: Methylprednisolone OND: Ondansetron; THD: Thalidomide; QOL: Quality of life; KPS: Karnofsky Performance Status; ECOG: The Eastern Cooperative Oncology Group; A: Acute phase; D: Delayed phase; O: Overall phase; AEs: Adverse Effects; NR: Not Reported

**Table 2:** Adverse events comparison between thalidomide and control group

Adverse Effects	Included studies	T+C			C			Heterogeneity analysis			Statistical analysis model		Statistical analysis	
		n	N		n	N		I²	p				OR(95%CI)	P
Myelosuppression	8	110	297		115	304		0.00%	0.94		Fixed effect model		0.98(0.69-1.39)	0.89
Constipation	27	547	1288		366	1246		36.20%	0.06		Fixed effect model		1.84(1.54-2.19)	<0.01
Drowsiness/Dizziness	21	324	938		151	861		66.80%	<0.01		Fixed effect model		2.67(2.14-3.34)	<0.01
Sedation	4	81	437		29	443		21.20%	0.28		Fixed effect model		3.37(2.15-5.31)	<0.01
Rash	14	76	595		55	527		55.60%	0.02		Fixed effect model		1.31(0.91-1.89)	0.15
Diarrhea	6	60	606		41	589		0.00%	0.85		Fixed effect model		1.44(0.94-2.19)	0.09
Peripheral neuropathy	14	85	531		55	504		0.00%	0.58		Fixed effect model		1.58(1.14-2.18)	0.06
Hepatorenal damage	10	64	358		59	354		0.00%	0.97		Fixed effect model		1.07(0.72-1.58)	0.74

Abbreviations: T+C: Thalidomide group; C: Control group; n: number of patients have adverse events; N: number of patients allocated to the two groups
